# Editorial: Wnt signaling in endocrine and metabolic disorders

**DOI:** 10.3389/fendo.2023.1254977

**Published:** 2023-08-07

**Authors:** Frederic Cailotto, Gaetano Santulli

**Affiliations:** ^1^ UMR7365 Ingénierie Moléculaire et Physiopathologie Articulaire (IMOPA) Centre National de la Recherche Scientifique - Université de Lorraine (CNRS-UL), Biopôle de l’Université de Lorraine, Vandœuvre-lès-Nancy, France; ^2^ Department of Medicine, Wilf Family Cardiovascular Research Institute, Fleischer Institute for Diabetes and Metabolism (FIDAM), Albert Einstein College of Medicine, New York, NY, United States; ^3^ Department of Molecular Pharmacology, Einstein-Mount Sinai Diabetes Research Center (ES-DRC), Einstein Institute for Aging Research, Institute for Neuroimmunology and Inflammation (INI), Albert Einstein College of Medicine, New York, NY, United States

**Keywords:** Wnt, metabolism, endocrinology & metabolism, Wnt/b-catenin, endocrinology, microRNA, Alzheimer’s disease, cancer

The present Research Topic, entitled “*Wnt signaling in endocrine and metabolic disorders*” aims to emphasize the functional role of the Wnt signaling pathway in human endocrinology, focusing on metabolic disease. Endocrine and metabolic disorders encompass a wide range of conditions affecting various organ systems and physiological processes. The Wnt signaling pathway, originally recognized for its role in embryonic development and tissue homeostasis (1, 2), has emerged as a crucial player in the pathogenesis of several human disorders, including cancer (3, 4), and greatly contributes to disease progression and potential therapeutic implications (5–7).

The first study in this Research Topic clarified that one of the mechanisms by which the “*Modified Qing’ E Formula*” (MQEF), used for more than 1,300 years in China as a treatment for lumbodynia, may exert its therapeutic effect on steroid-induced ischemic necrosis of the femoral head, is through targeting exosomal microRNAs (miRNAs) to regulate multiple signaling pathways, including Wnt, PI3K-Akt, and MAPK (Zhu et al.). In another original report investigating miRNAs and Wnt signaling, Tripathi et al. demonstrate that miR-539-3p overexpression in osteoblasts downregulates several components of the Wnt signaling pathway and deteriorates trabecular microarchitecture, leading to decreased bone formation in ovariectomized mice. In the third original article in our Research Topic, a group of investigators led by Xiaolin Tu found that the small molecule C91 (CHIR99021) promotes osteogenic differentiation of bone marrow stromal cells *via* the activation of Wnt signaling (Wang et al.).

Vilaseca et al. provide an interesting overview of the functional roles of estrogen deficiency in the processes involved in the development of Alzheimer’s disease, including Wnt signaling and glucose transport in the brain, amyloid precursor protein processing to form senile plaques, and Tau phosphorylation forming neurofibrillary tangles.

A very comprehensive review concludes our Research Topic: Franco et al. elegantly explain the main differences between the physiological roles of canonical Wnt signaling (essential for cell growth, tissue remodeling, and organ formation) and its pathological involvement in the development of several human diseases, including cancer. Correctly interpreting the molecular bases of Wnt signaling and metabolism, ideally in a cell-type and tissue-specific manner (Franco et al.; 8–10), may provide formidable knowledge to biomedical scientists and clinicians, holding the promise of producing novel therapies.

In conclusion, understanding the intricate interplay between Wnt signaling and endocrine/metabolic disorders holds great promise for the development of targeted therapies and improved patient outcomes.

## Author contributions

FC: Writing – review & editing. GS: Writing – original draft
